# Test Learning Effects Influence Coordinative but Not Strength-Related Tasks in Patients Six Months After Anterior Cruciate Ligament Reconstruction

**DOI:** 10.3390/jcm14176308

**Published:** 2025-09-06

**Authors:** Sonja Jahnke, Robert Prill, Aleksandra Królikowska, Łukasz Oleksy, Caren Cruysen, Maciej Daszkiewicz, Mateusz Kowal, Monika Kentel, Maciej Kentel, Sven Michel, Paweł Reichert, Roland Becker

**Affiliations:** 1 Center of Orthopaedics and Traumatology, University Hospital Brandenburg/Havel, Brandenburg Medical School Theodor Fontane, 14770 Brandenburg an der Havel, Germany; caren.cruysen@mhb-fontane.de (C.C.); roland.becker@mhb-fontane.de (R.B.); 2 Faculty of Health Sciences Brandenburg, Brandenburg Medical School Theodor Fontane, 14770 Brandenburg an der Havel, Germany; 3 Physiotherapy Research Laboratory, University Centre of Physiotherapy and Rehabilitation, Faculty of Physiotherapy, Wroclaw Medical University, 50-372 Wroclaw, Poland; aleksandra.krolikowska@umw.edu.pl (A.K.); daszkiewiczmaciej98@gmail.com (M.D.); mateusz.kowal@umw.edu.pl (M.K.); 4 Department of Orthopaedics, Traumatology and Hand Surgery, Faculty of Medicine, Wroclaw Medical University, 50-556 Wroclaw, Poland; loleksy@oleksy-fizjoterapia.pl (Ł.O.); pawel.reichert@umw.edu.pl (P.R.); 5 Department of Physiotherapy, Faculty of Health Sciences, Jagiellonian University Medical College, 31-008 Kraków, Poland; 6 eMKa MED Medical Center, 53-110 Wroclaw, Poland; monika.kentel@emkamed.com.pl (M.K.); maciej.kentel@emkamed.com.pl (M.K.); 7 Department of Therapy Sciences, Brandenburg University of Technology Cottbus-Senftenberg, 01968 Senftenberg, Germany; sven.michel@b-tu.de

**Keywords:** anterior cruciate ligament reconstruction (ACLR), Return to Sport (RTS), Back in Action (BIA), coordination, neuromuscular adaption, kinematics

## Abstract

**Background**: A comprehensive rehabilitation program is recommended following anterior cruciate ligament reconstruction (ACLR) to restore function, strength and lower limb stability. Despite advancements in surgical techniques, high reinjury rates highlight the need to refine rehabilitation strategies. This study investigates performance across various physically demanding tasks and compares outcomes between the injured and uninjured leg, using a modified Back in Action (BIA) test battery. It is hypothesized that due to test learning effects patients exhibit faster improvement in coordinatively demanding tasks compared to strength-related tasks. **Methods**: Thirty-two patients (aged 18–40) following primary unilateral ACLR participated in a prospective cross-sectional study within the context of a larger trial. Participants completed a modified BIA test battery, including stability, countermovement jump (CMJ), drop landing, speedy jumps, and quick-feet test (QFT). Each test was conducted in three sets, with three immediate repetitions. Self-reported outcomes were also collected. **Results**: Patients showed significant within-session improvements in coordinative tasks, with 32% in the injured and 26% in the uninjured limb during the speedy jumps [*p* < 0.05]. No significant learning effects were observed in strength-related tasks (drop landing, stability test, CMJ). Patients with lower baseline performance exhibited greater improvement than patients with higher performance level from baseline. Furthermore, a correlation between self-assessed abilities and actual performance was identified. **Conclusions**: This study showed that improvement of coordinative tasks after Return to Sport (RTS) testing of ACLR patients are rather affected by test learning effects. In contrast, this phenomenon is not seen in strength-related tasks. Rehabilitation programs should integrate both types of exercises while considering individual baseline abilities. Tailoring rehabilitation to individual needs, establishing sport-specific rehabilitation programs and incorporating self-assessment tools can enhance patient-centered care and reduce reinjury risks.

## 1. Introduction

Recovery following anterior cruciate ligament reconstruction (ACLR) and achieving a safe Return to Sport (RTS) remains challenging. Despite advancements in surgical techniques, young athletes, in particular, continue to experience high reinjury rates, highlighting the need to evaluate and improve current rehabilitation strategies [1,2,3]. A carefully tailored rehabilitation program is essential for enabling patients to return to their preinjury level of athletic activity and for reducing the risk of reinjury and secondary complications, such as an early onset of osteoarthritis or meniscal tears [4,5].

Determining the optimal timing for RTS is complex, as it involves multiple physical, psychological, and sprot-specific factors, and there is currently no gold standard [6,7]. Additionally, identifying individual prognostic factors for re-rupture [8,9] or primary contralateral ACL injury remains challenging. Therefore, RTS decisions should involve a multidisciplinary team, including the patient, surgeon, team physician, physical therapist, and athletic trainer [10].

Test batteries are commonly used to gain prognostic insight into a patient’s performance, assessing strength, stability, proprioception, speed, and movement quality [11,12,13]. These assessments should reflect sport-specific demands, be practical for daily implementation, and accurately monitor functional progress. One common test battery, the Back in Action test battery (BIA), is a validated, standardized tool that is both practical and efficient [14]. It assesses seven functional tests and may help in the RTS decision-making process.

It is essential to define individualized rehabilitation goals, taking into account each patient’s personal aspirations, sporting background, performance level, and functional capacity. Rather than relying on time-based benchmarks, RTS decisions should be guided by performance-based assessments [10,15].

Digital health applications are increasingly used to support rehabilitation by supplementing physiotherapy sessions in a time- and location-independent manner [16]. Regular postoperative use has been associated with improved outcomes, including reduced extension deficits and a lower incidence of cyclops syndrome [17,18].

A multimodal approach that incorporates coordinative, strength, and stability-based exercises has been shown to improve recovery outcomes. While strength-based tasks preliminary focus on rebuilding physical strength and endurance, coordinative tasks help regain motor control. Proprioceptive and stability exercises significantly improve joint position sense and perceived knee function [19]. Most exercises involve a blend of multiple physical demands, with one component typically being more dominant. It remains unclear which abilities can recover faster, and which require more time.

The Limb Symmetry Index (LSI) is a common parameter to evaluate the readiness for RTS. Research has shown sustained or even increasing deficits in muscle strength, sensorimotor function, and functional performance in the injured limb. Giacomazzo et al. highlight persistent asymmetries and functional limitations in Single-leg Vertical Jump and Drop Jump Performance 7 months after ACLR [20]. Similarly, Kotsifaki et al. reveal that athletes continue to show asymmetry during vertical jumps, particularly in the concentric phase and during bilateral jump landings [21].

Evidence indicates that patients with strong baseline physical performance tend to recover more quickly after ACLR than those with lower initial performance levels. Consequently, it is valuable to analyze test improvements among patients with varying athletic backgrounds and to compare their immediate, individualized progress when repeating exercises.

The primary aim of this study was to assess short-term performance improvements in coordinative, time-based tests (speedy jumps, quick-feet test), stability and strength-related tests (stability test, countermovement jump, drop landing) using a modified BIA test battery, six months after ACLR.

It was hypothesized that, due to test learning effects, patients exhibit faster improvement in coordinatively demanding tasks compared to strength-related tasks.

The analysis of in-session test improvements among patients with varying athletic backgrounds will provide insights into motor learning effects in terms of RTS test batteries. These findings could support the development of more individualized rehabilitation strategies for ACLR patients. Additionally, the influence of baseline performance levels and patient-reported outcome measures were examined to provide a more comprehensive perspective on recovery progression.

## 2. Materials and Methods

This study was conducted as a prospective cross-sectional analysis within the framework of a larger prospective cross-over clinical trial and performed at the Trauma and Orthopedic Department eMKa Med Medical Center in Wroclaw, Poland. Thirty-two patients who underwent primary unilateral ACLR six months (±1 month) prior were recruited via registry.

Ethical approval for this study was granted by the district medical chamber as an extension of a previously approved ethical application (AS 107(dB)/2018). Informed consent and confirmation of personal data protection were obtained from all participants. Patient anonymity was maintained, with data re-identifiable only through the hospital’s security system. Patients were informed about the performance but not about the specific objectives of these tests. While patients’ performance was also evaluated in terms of the influence of braces’ usage, for this study only the influence of test repetition was assessed. Patients did not follow a uniform rehabilitation program at a single center. Rehabilitation protocols varied depending on the treating clinician and local resources, reflecting real-world clinical practice. To ensure comparability at the time of testing, all participants met the same inclusion and exclusion criteria.

Inclusion criteria: age 18–40 years; six months (±1 month) after post-traumatic primary unilateral ACLR, with the use of the autologous ipsilateral semitendinosus graft; body mass index (BMI) ≤ 24.9 kg/m^2^; moderate activity with regard to the rehabilitation stage and frequent recreational sporting activity prior to the ACL injury.

Exclusion criteria: additional procedures including meniscus sutures; osteoarthritis; history of injury or disease in the ACLR limb prior to the ACL injury; history of injury or disease in the adjacent joints, contralateral limb, or spine within the last 6 months; secondary ACLR or current permanent knee pain or swelling.

**Figure 1:**
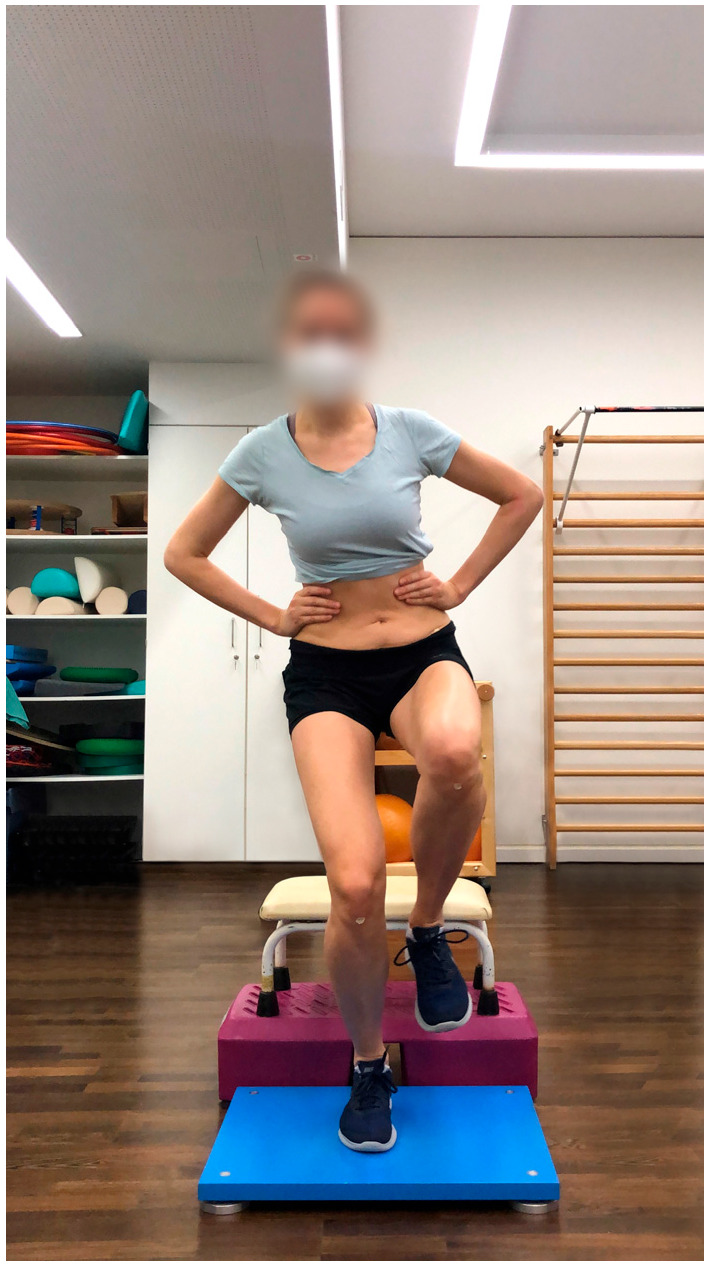
Stability test.

**Figure 2:**
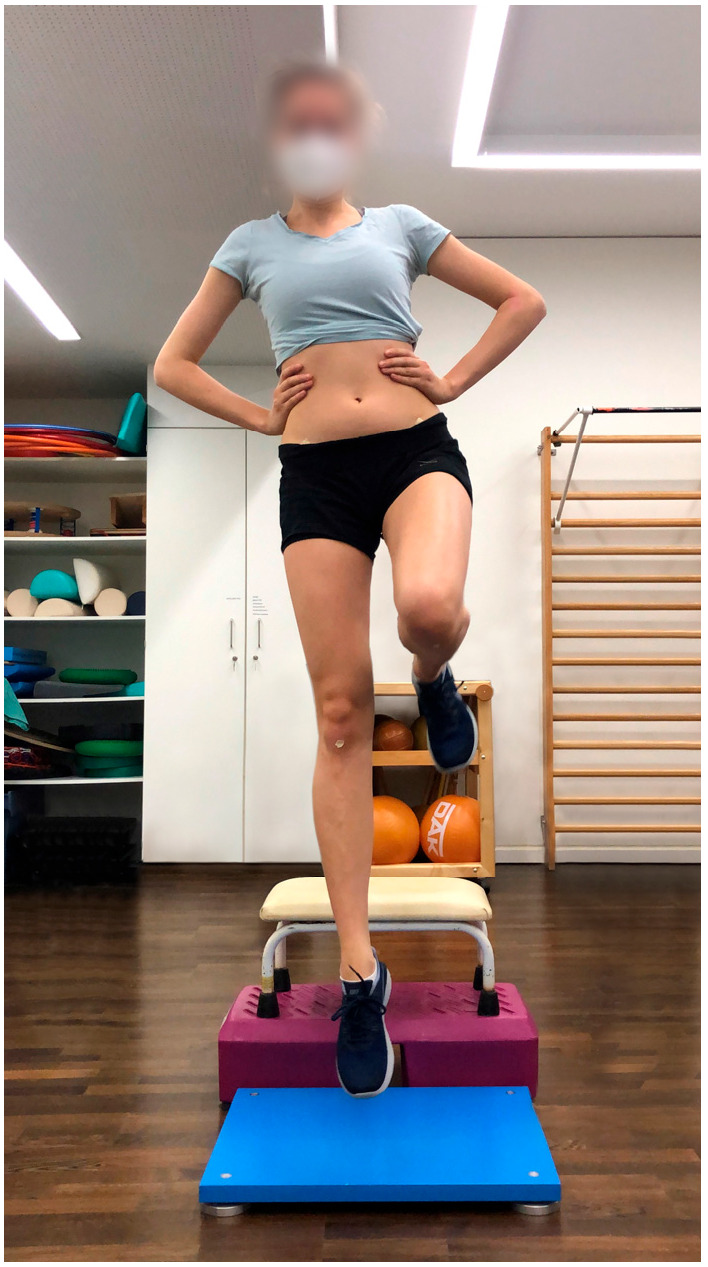
CMJ.

**Figure 3:**
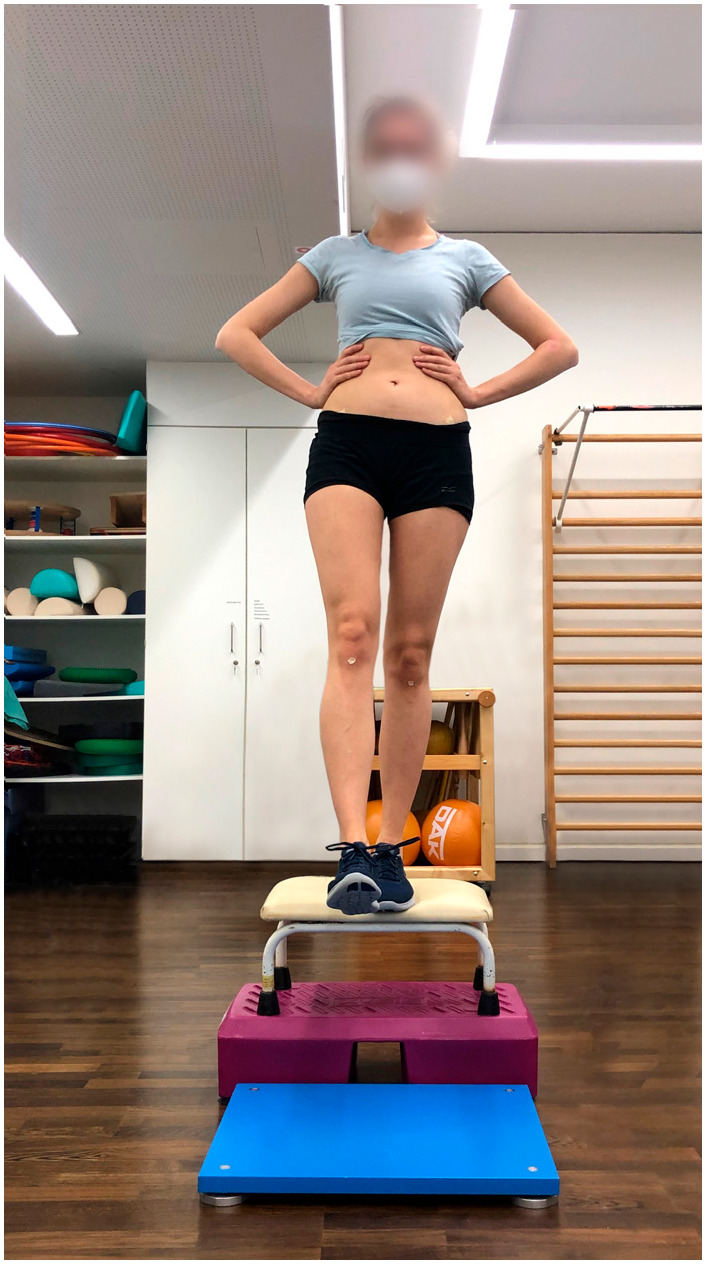
Drop landing.

**Figure 4:**
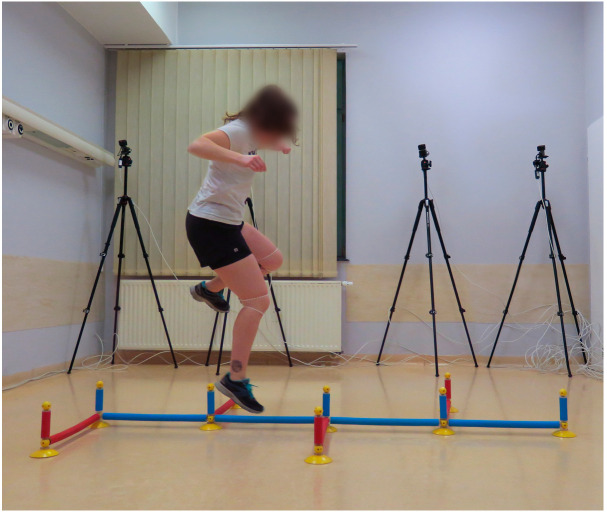
Speedy jumps.

**Figure 5:**
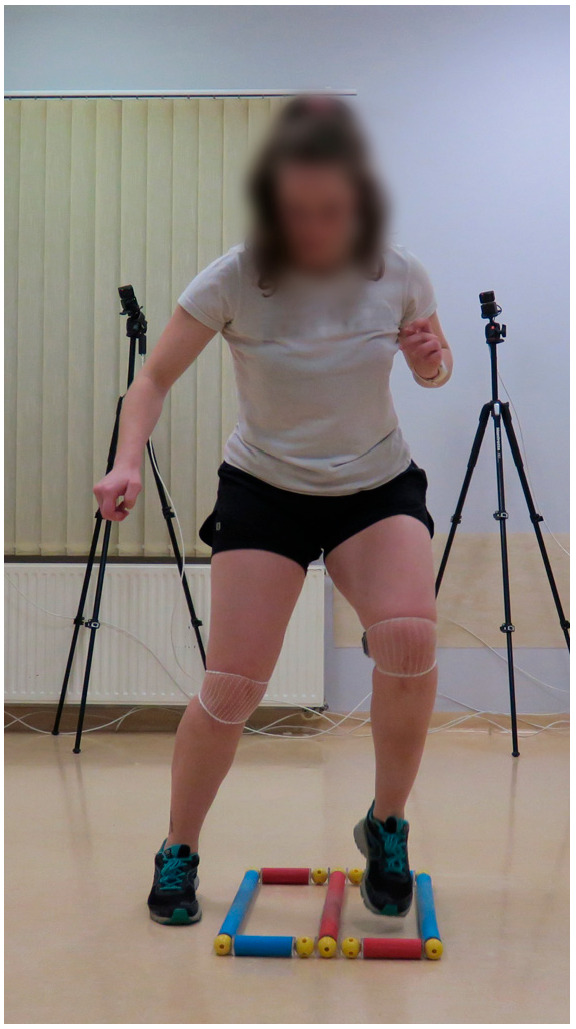
QFT.

Procedure: This modified BIA test battery consists of seven functional performance tests to assess stability, strength, coordination, and speed. The full study protocol for the BIA test battery, along with detailed procedures for each test, has been previously published [22]. For this study, individual elements of the test battery were analyzed separately. Participants started with a five-minute warm-up on the cycle ergometer at 75 watts, followed by three sets of five squats, and then completed the test battery:

- Single-leg stability test: The test was performed using a 3D force plate, with patients balancing for 10 s on each leg (Figure 1).
- Single-leg countermovement jump (CMJ): Starting from a semi-squat position on one leg, patients performed an explosive jump, aiming to reach maximum height as quickly as possible before landing back on the same leg in a controlled semi-squat (Figure 2).
- Single-leg drop landing: Patients stepped off a 40 centimeter vaulting box onto the force plate, holding the position for five seconds without losing balance (Figure 3).
- Single-leg speedy jumps: Patients completed 16 single-leg jumps through red (forward–backward–forward jumps) and blue (sideways jumps) hurdles in a coordination parkour as quickly as possible, with time recorded [7] (Figure 4).
- Quick-feet test (QFT): Patients completed 15 steps in and out with one foot at a time, using their arms for balance, with time and tapping rate recorded [7] (Figure 5).

Each test was conducted in three sets, with each test repeated three times in immediate succession, always starting with the uninjured leg. The time interval between Set 1 and Set 3 was exercise-dependent but never exceeded 10 min. The best repetition from each set was selected for analysis. All tests were thoroughly explained and demonstrated by the same examiner, who consistently guided the participants throughout. Biokinematic data were recorded using the 3D force plate Kistler model 9260AA and analyzed by the performance analysis software MARS (Measurement, Analysis and Reporting Software 2875A3/A4) for Power and Strength, Balance, and Stability. The speedy jumps and QFT was executed using the Speedy Basic Jump Set (TST Trendsport, Grosshöflein, Austria).

Furthermore, patients completed the International Knee Documentation Committee (IKDC) questionnaire, the ACL-Return to Sport Injury Scale (ACL-RSI), and the Tegner Activity Scale (TAS). These patient-reported outcomes on symptoms, recovery, and knee functionality are of paramount importance, as they provide valuable subjective insights that complement the objective biomechanical assessments.

## 3. Statistical Analysis

Descriptive statistics were used to summarize the demographics of the patient population, including mean and standard deviation (SD) reporting for continuous variables. A Shapiro–Wilk test was performed to ensure that the data were normally distributed and suitable for linear correlation analysis. To evaluate the changes in the measurements between the first and third set, a paired *t*-test was conducted, allowing for the determination of statistically significant differences in mean values across the two time points within the same group of patients. Pearson correlation analysis was performed to assess the linear relationship between two continuous variables. Spearman’s rank correlation, as a non-parametric was employed to measure the strength and direction of association between two ranked variables. All statistical analyses were performed using IBM SPSS Statistics 29.0.2.0 and Microsoft Excel 2021, with a significance level set at [*p* < 0.05].

## 4. Results

Table 1 presents patient demographics and self-reported outcomes from the questionnaires. Values are reported as mean ± SD (range), unless otherwise noted.

**Figure 6:**
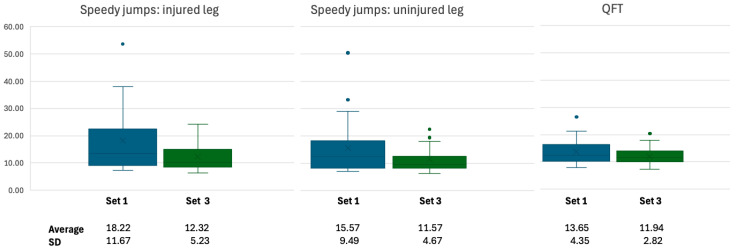
Test improvement (sec.) in coordinatively demanding tasks.

Patients exhibited significantly faster improvement in coordinatively demanding tasks compared to strength-related tasks (Table 2, Figure 6). The highest improvement was observed in the speedy jumps with the injured leg (32%), followed by the speedy jumps with the uninjured leg (26%) and the QFT (13%). The decrease in SD across these three tests indicates that patients not only performed faster but also more consistently in set three compared to set one. The Pearson correlation coefficients, all above 0.8, suggest a strong positive correlation between performances in the two sets, while the statistically significant *p*-values from the t-tests confirm the reliability of these improvements.

In contrast, performance in strength-related tasks such as CMJ, drop landing, and the stability test show varying degrees of changes between set one and set three across the different tests and limbs, but none of these changes reached statistical significance as indicated by the t-test results. The Pearson correlation coefficients generally suggest moderate positive relationships between the sets, except for the sway area in the uninjured leg, which showed a weak correlation.

When comparing limbs, the injured leg exhibited significantly greater improvement in the speedy jumps, with a 32% reduction in time compared to a 26% reduction for the uninjured leg (Table 2, Figure 6). However, in tasks like stability test and drop landing, the uninjured leg showed some improvement (5.42% and 74.61%, respectively) while the injured leg displayed negative or minimal changes (−3.76% and −3.33%, respectively). In the CMJ, the injured leg showed a 25% decrease in performance, whereas the uninjured leg exhibited a 10% improvement.

**Figure 7:**
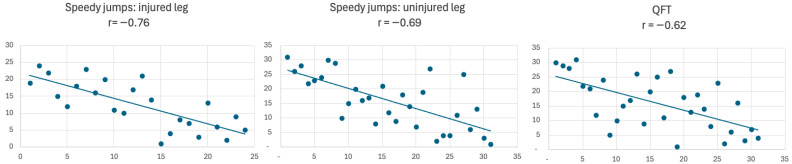
Inverse correlation between baseline performance and improvement in speedy jumps and QFT.

Further analysis explored the relationship between baseline performance and the degree of improvement. The results revealed a strong inverse correlation, indicating that patients with initially lower performance in the first set (higher rank) tended to show greater improvement in the third set (lower rank) (Figure 7). Conversely, patients with higher baseline performance in set one exhibited smaller improvement in performance in the third set. This is evidenced by strong negative Pearson correlations: r = −0.76 for the injured leg in the speedy jumps, r = −0.69 for the uninjured leg and r = −0.62 for the QFT.

**Figure 8:**
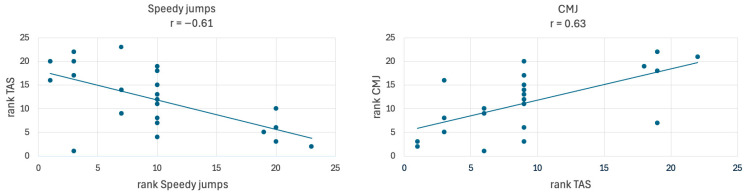
Correlation between TAS and demonstrated performance of speedy jumps and CMJ.

Finally, the scatter plots illustrate the correlation between patients’ perceived and demonstrated performance in both the speedy jumps (r = −0.61) and CMJ (r = 0.63) (Figure 8). In the speedy jumps, faster performance (lower ranks) was associated with a higher TAS, as reflected by the negative correlation. Similarly, patients who achieved higher values in the CMJ tended to have higher ranks in the TAS, indicating a positive correlation between perceived and demonstrated jump performance.

## 5. Discussion

Based on the results, the hypothesis can be accepted, suggesting that patients tend to show a more rapid improvement in coordinatively demanding tasks (speedy jumps and QFT) compared to strength-related tasks (CMJ). These findings support the idea that coordination skills are determined by the regulation of movement, which involves processes such as receiving, processing, and storing perceptual, cognitive, and memory-related information. They are characterized by qualities such as speed, accuracy, flexibility, and economy [23]. Tasks such as single-leg speedy jumps require the integration, synchronization, and coordination of multiple aspects, such as timing, jump height, landing control, balance, and speed. Those aspects are predominantly influenced by coordinative related abilities. The quick adaptation observed in coordinative tasks might be attributed to motor control improvement and learning effects, resulting in a mix of adaptation in terms of feedforward and feedback strategies. Feedforward strategies anticipate future events based on past experiences, while feedback strategies adjust actions based on responses to immediate stimuli [24]. The rapid improvement in coordination tasks can be attributed to these learning mechanisms and the central nervous system’s ability to quickly adapt to movement patterns and coordination demands.

In contrast, strength-related tasks such as the CMJ rely more on muscular strength, power and endurance—attributes that require longer periods of physical training to achieve measurable improvement.

These findings suggest that coordinative abilities can be improved faster than strength, which may inform the design of rehabilitation planning. Programs may benefit from placing greater emphasis on strength-related exercises to support long-term physical adaption, while ensuring consistent integration of motor coordination training.

Interestingly, the injured leg showed more significant improvements in dynamic coordinative tasks (speedy jumps) compared to the uninjured leg. However, in balance-based tasks, no such superiority was observed. After an ACLR neuromuscular control and somatosensation are initially compromised, affecting the control of the skeletal muscle system and leading to altered peripheral muscle activation [25,26]. This may result in increased cognitive effort and faster adaption in the injured leg, as the nervous system adjusts faster to the task. Furthermore, psychological factors may play a role as the fear of reinjury could also contribute to more precise performance in the injured leg. Additionally, rehabilitation programs often emphasize targeted training for the injured leg, which may further accelerate improvement.

In conclusion, during the rehabilitation process, it is important to retrain both legs, as the injured leg may catch up or even surpass the uninjured leg due to its higher prioritization. This approach could help minimize the risk of reinjury caused by muscle imbalances.

The speedy jumps and the QFT results indicate that patients with initially lower baseline performance exhibited more significant improvements compared to stronger patients. One key factor may be that strong individuals might already be closer to their maximum performance level, leaving less room for improvement during immediate test learning effects. In contrast, patients with lower performance start from a lower baseline and therefore profit immediately from practicing a certain task. Therefore, as in most sporting activities it impresses more challenging to improve a strong performance than a weaker one, which may result in fewer repetitions yielding greater progress in patients with lower initial performance levels. Furthermore, the disparity in performance even six months post-ACLR may reflect different stages of neuromuscular adaptation. Strong patients may already be well-adapted to such tasks, so additional practice leads to relatively smaller improvements. However, patients with lower baseline performance may show faster progress in coordination and efficiency, leading to more significant learning effect. Prehabilitation before surgery has been shown to enhance early recovery outcomes after ACLR, as it helps to restore muscle strength and improves neuromuscular control [27,28,29,30,31].

At six months post-ACLR, patients demonstrated a notable decline in activity levels as measured by the TAS (Table 1). The preinjury mean TAS was 6.27 ± 1.48, decreasing to 4.4 ± 1.70 at six months postoperatively. These findings indicate that patients had not yet returned to their previous level of sports participation, emphasizing that RTS decisions should rely on performance-based assessments rather than solely on time-based benchmarks.

Furthermore, a correlation between patient’s self-assessment scores (TAS) and their actual performance in the speedy jumps and CMJ was observed. Patients who performed well in those dynamic, high-demand tasks tended to rate their abilities more favorably, suggesting that their subjective evaluations corresponded closely with their objective performance. Therefore, the TAS seems to effectively evaluate these abilities and may play an important role in the RTS decision making process.

When conducting a test battery, it is essential to understand the characteristics of each test and carefully consider the validity of each test for the ability of interest. This includes not only the classic quality criteria such as validity, reliability, and objectivity but also factors like the test’s discriminatory power, difficulty level, efficiency, and comparability. When assessing validity, it is crucial to consider which ability should be tested. Most commonly general coordinative ability models are used to identify ability or disability of interest [23].

Additionally, one should consider whether repeated testing may be influenced by learning effects, potentially reflecting improvements that are not directly related to anatomical or functional progress in the patient. Neumaier and colleagues propose focusing not only on categorizing a patient’s ability but also on analyzing the demands of the tasks themselves. Therefore a structural model that includes information requirements and pressure conditions is used [32]. The information requirements refer to afferent information from various sensory organs (visual, auditory, kinesthetic, vestibular, and tactile), along with the integration of these sensory inputs. The pressure conditions encompass precision pressure, time pressure, complexity pressure, situational pressure, and exertion-related stress. The combination of these movement- and situation-related factors determines the coordination challenge and the profile required for the task. It is crucial to tailor the individual criteria to the specific demands of the sport, focusing on typical, performance-relevant situations and key actions to ensure targeted and effective training [32,33,34].

**Figure 9:**
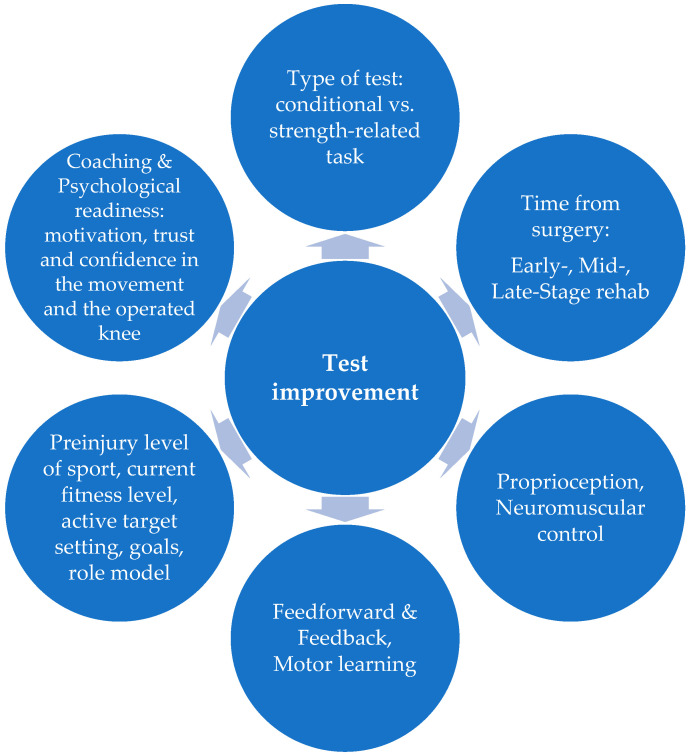
Factors influencing test performance improvement—a multidimensional approach.

Based on this explanation, it is advisable to design a test with as few variables as possible to ensure the test’s appropriateness. However, when evaluating an improvement in test performance, other factors that cannot be directly controlled should also be considered. These include, e.g., the patient’s preinjury level of sport, their use of feedforward and feedback strategies, individual motor learning speed, motivation, attitude, and time since the surgery. Psychological readiness, in particular, is a critical determinant for RTS and should be routinely assessed [35,36,37,38] (Figure 9).

Ultimately, the results of repeated testing should be interpreted cautiously, as observed improvements may be driven by motor learning or familiarization with the task rather than true functional recovery.

By considering these nuances, clinicians can more effectively monitor patient progress, tailoring rehabilitation programs to the unique demands.

Some limitations should be considered when interpreting the findings of this study. The results of this research demonstrated improvements in the targeted skills, but no follow up measurement for assessing the sustainability of improvements was performed. However, analyzing the immediate effects is valuable as it gives initial insights into the efficacy and can be used as a baseline for further research on this topic. Additionally, as the study design involved three repetitions of the sets, it cannot be ruled out that further repetitions could have led to a plateau in improvement or even a diminishment due to any kind of fatigue. Finally, the study took place in a controlled setting; therefore, the results might not be generalizable to all testing situations in clinical or training practice. Nevertheless, the controlled conditions allowed us to identify the mechanisms behind the observed improvement. Investigating how improvement develops under different neurocognitive stimuli (e.g., visual, haptic, acoustic) or environmental stressors might provide further insights into their practical applicability in a real-world scenario.

## 6. Conclusions

This study provides valuable insights into the rehabilitation process following ACLR, particularly in understanding the dynamics of improvement in coordinative vs. strength-related tasks. The findings highlight that coordinative tasks show more rapid short-term improvement compared to strength tests, which require structural adaptation. This highlights the importance of individual test assessments, as some tests might show improvements due to learning effects. Additionally, the observed greater improvement in the injured leg compared to the uninjured leg emphasizes the need for targeted rehabilitation strategies that address both limbs, potentially reducing the risk of reinjury due to muscle imbalances. The study also revealed that patients with initially lower performance tend to exhibit greater gains, suggesting that rehabilitation programs should be tailored to accommodate individual baseline abilities. Both aspects show that more capacity for improvement leads to larger improvement during test repetition. Furthermore, the correlation between perceived and actual performance underscores the value of self-assessment tools like the TAS. Future research should explore the sustainability of the improvements and investigate the applicability of these findings in several environments. Overall, the study enhances our understanding of ACLR rehabilitation, providing a foundation for developing more effective and personalized rehabilitation strategies that can improve patient outcomes and reduce the risk of reinjury.

## Figures and Tables

**Table 1:** Patient demographics and Patient-Reported Outcome Measures (PROMs).

Age (years)	31.74	±8.47
Sex (F/M)	11/21	
Body height (m)	1.76	±0.07
Body mass (kg)	72.55	±10.76
Body mass index	22.09	±2.81
TAS preinjury	6.27	±1.48
TAS 6m post-op	4.4	± 1.70
ACL-RSI (%)	51.67	± 24.29
IKDC (%)	68.82	± 14.95

TAS = Tegner Activity Scale; ACL-RSI = ACL-Return to Sport Injury Scale; IKDC = International Knee Documentation Committee.

**Table 2:** Performance metrics and statistical measures of injured and uninjured leg across various functional tests, alongside mean, standard deviation (SD), Pearson correlation coefficients, *p* (*t*-Test, two tailed), Cohen’s *d*, and percentage changes in performance between the sets; COP = center of pressure.

	Speedy Jumps Injured Leg		Speedy Jumps Uninjured Leg		QFT	
	Set 1	Set 3	Set 1	Set 3	Set 1	Set 3
Mean (s)	18.22	12.32	15.57	11.57	13.65	11.94
SD	11.88	5.26	9.49	4.67	4.35	2.82
Pearson r	0.904		0.842		0.886	
*p* (*t*-Test, two tailed)	0.00061		0.00049		0.00010	
Cohen’s *d*	0.64		0.53		0.47	
Improvement (%)	32		26		13	
**Single-Leg CMJ**	**Injured Leg**			**Uninjured Leg**		
	**Set 1**		**Set 3**	**Set 1**	**Set 3**	
Mean (Jump height from take off in m)	0.08		0.06	0.10	0.11	
SD	0.12		0.05	0.08	0,07	
Pearson r	0.393			0.329		
*p* (*t*-Test, two tailed)	0.786			0.657		
Cohen’s *d*	0.22			−0.13		
Change (%)	−25			10		
**Single-Leg Drop Landing**	**Injured Leg**			**Uninjured Leg**		
	**Set 1**		**Set 3**	**Set 1**	**Set 3**	
Mean (stability time from COP in sec.)	1.86		1.79	1.66	1.75	
SD	1.09		0.82	1.07	1.07	
Pearson r	0.377			0.439		
*p* (*t*-Test, two tailed)	0.754			0.877		
Cohen’s *d*	0.07			−0.08		
Change (%)	−3.76			5.42		
**Single-Leg Stability Test**	**Injured Leg**			**Uninjured Leg**		
	**Set 1**		**Set 3**	**Set 1**	**Set 3**	
Mean (Sway area—total [mm^2^])	4503		4353	4808	8395	
SD	3032		3430	3109	17016	
Pearson r	0.393			0.128		
*p* (*t*-Test, two tailed)	0.786			0.255		
Cohen’s *d*	0.05			−0.29		
Change (%)	−3.33			74.61		

## Data Availability

Data available on request due to the privacy and ethical requirements: The data presented in this study are available on request from the corresponding authors. The data are not publicly available due to privacy or ethical restrictions.
